# The utilization of data analysis techniques in predicting student performance in massive open online courses (MOOCs)

**DOI:** 10.1186/s41039-015-0007-z

**Published:** 2015-07-16

**Authors:** Glyn Hughes, Chelsea Dobbins

**Affiliations:** School of Computing and Mathematical Sciences, Liverpool John Moores University, Byrom Street, Liverpool, L3 3AF UK

**Keywords:** Open learning, Prediction, Data analysis

## Abstract

The growth of the Internet has enabled the popularity of open online learning platforms to increase over the years. This has led to the inception of Massive Open Online Courses (MOOCs) that globally enrol millions of people. Such courses operate under the concept of open learning, where content does not have to be delivered via standard mechanisms that institutions employ, such as physically attending lectures. Instead learning occurs online via recorded lecture material and online tasks. This shift has allowed more people to gain access to education, regardless of their learning background. However, despite these advancements, completion rates for MOOCs are low. The paper presents our approach to learner predication in MOOCs by exploring the impact that technology has on open learning and identifies how data about student performance can be captured to predict trend so that at risk students can be identified before they drop-out. The study we have undertaken uses the eRegister system, which has been developed to capture and analyze data. The results indicate that high/active engagement, interaction and attendance is reflective of higher marks. Additonally, our approach is able to normalize the data into consistent a series so that the end result can be transformed into a dashboard of statistics that can be used by organizers of the MOOC. Based on this, we conclude that there is a fundamental need for predictive systems within learning communities.

## Introduction

The evolution of technology, and ease of communication through the Internet and World Wide Web (WWW) has dramatically altered the landscape of teaching and learning in higher education (Kop, 2011). In its infancy, the first iteration of the WWW (Web 1.0) was simply a place for users to gather information, from static web pages, to supplement their learning and offered very little communicative capabilities (Nath et al. 2014). However, the inception of Web 2.0 provided a new platform where users could read, write, modify, and update content online (Nath et al. 2014). This development enabled users to become active participants of the web and has allowed technologies and websites, such as blogs, YouTube, and wiki’s to be at the forefront of the user’s learning experience (Duffy 2008). As technology develops and more devices become connected, the convergence of people, process, data, and things will enable the Internet of Everything (IoE) to be the next trend of the Internet’s evolution (Bradley et al. 2013). This rapid growth has created a $14.4 trillion market and has seen approximately over 10 billion devices being connected to the Internet, with this number set to increase to 50 billion by 2020 (Bradley et al. 2013). As such, the IoE will enable educational institutions to be available to people who previously did not have access and will improve a number of issues, including (1) access to content by addressing scalability issues so that course material and recordable instructions can be available on any device, at any time, (2) improved quality of learning by enabling people to access and study material at their own pace, and (3) the ability to access proactive content, free materials, and customization of curriculum (Bradley et al. 2013). This shift of instant connectivity has produced a new type of student who now have the option of learning online, without having to formally attend an institution, and who are experiencing education in different ways. This phenomenon is known as e-learning and can be described as a new framework for education whereby considerable amounts of information, which describe a variety of teaching–learning interactions, are endlessly generated and ubiquitously available (Castro et al. 2007).

One outcome of this improved connectivity are MOOCs, which are quickly developing as a popular way for a wide range of communities, who may not have access to an institution, to become involved in online distance education (Clarà and Barberà 2013). Through such high-profile platforms, including Coursera, EdX, and Udacity, free courses have become available from a range of exclusive universities, which is altering the way people are undertaking learning (Jordan 2014). Furthermore, the benefit of instantly accessing high-quality educational material, regardless of location and educational background, has attracted a large range of students onto these courses (Balakrishnan and Coetzee 2013). As such, the development of large-scale MOOCs has increased over the years, with enrolment on such courses averaging around 33,000 students (Jordan 2014). Nevertheless, whilst enrolment is quite high, only 7.5 % of students complete their course, with the main reason for withdrawal being attributed to poor time management skills (Jordan 2014; Nawrot and Doucet 2014). In order for MOOCs to have an impact in the educational sector, maintaining and supporting student engagement are a necessity (Ramesh et al. 2013). In order to alleviate this issue, to a certain extent, data analytic techniques can be used to study student engagement with their course in order to identify and predict trends about a student’s performance. This is important as engagement is positively linked to academic performance (Carini et al. 2006). By providing this information to the student at an early stage, it is hoped that this will serve as a motivational tool to improve. As described by Simpson (2006), predicting student success in distance education is particularly important for new students as the pre-course information is sometimes inadequate and withdrawal often occurs very early. Measures such as sex, previous educational qualifications, and age have been used in logistic regression analysis to identify a new student’s chance of withdrawing (Simpson 2006). However, analysis of engagement with course material, via learning management systems (LMSs), offers a considerable amount of more information that is very valuable for analyzing behavior and predicting success (Romero et al. 2013).

Globally, data has increased substantially over the past 20 years, with hundreds of petabytes (PB) being processed monthly (Chen et al. 2014). This growth of information can be attributed to the medium of Web 2.0 services, the IoE, social networks, medical applications, online education services, and cloud computing; data is everywhere and in every sector (Chen et al. 2014). As such, the term “big data” is often used to describe datasets that have grown in size well beyond exabytes and zettabytes. These datasets reach a point where the ability to capture, manage, and process such items, within a reasonable amount of time, cannot be achieved with commonly used software tools (Kaisler et al. 2013; Wu et al. 2014). This type of data can be characterized by the four V’s—volume, variety, velocity, and veracity. Volume relates to the amount of data that an organization can access but not necessarily own (e.g., social media and IoE). Variety pertains to the richness of the data that has been obtained from multiple sources (text, images, video, audio, etc.). Velocity is the speed at which data is created, streamed, and aggregated, whilst veracity relates to the accuracy of the data (Kaisler et al. 2013; O’Leary 2013). In terms of MOOCs, a variety of information can be gathered about a student to indicate engagement with their course, including engagement with online course materials, communication with the online community by posting in forums and asking and answering questions or by watching lectures and taking quizzes, without such interaction (Ramesh et al. 2013). This data can then be used to profile them and predict their performance. As these courses gain popularity, a concern in this new era of data generation and open learning is the rapid extraction of vital and valuable information from such big datasets that can be used to the benefit of people and institutions (Chen et al. 2014). However, the application of data analysis and mining techniques can be used to overcome this problem. This area brings together the fields of statistics, pattern recognition, and machine learning to extract knowledge and detect patterns from complex sets of data (Castro et al. 2007). In the case of MOOCs, such techniques can be used to analyze student-generated data in order to find patterns of system usage and behavior, which can be used to indicate performance and predict trends (Castro et al. 2007). As such, educational data mining (EDM) has emerged as a field in itself to resolve such research issues (Romero and Ventura 2010).

With the advent of smarter devices, technology has become instrumental in the development of open learning and is widening the availability of such services to people who may have been previously restricted from the chance to enhance their education. As enrolment on MOOC’s increases and students generate more data, the pool of information that is available to obtain knowledge is becoming richer. This paper explores the impact of technology on opening learning and examines how data analytics can be used to identify and capture relevant data about student performance and engagement to predict trends.

## Background

The landscape of our environment is becoming more and more digital, with online learning and MOOCs becoming increasingly popular. Nevertheless, despite their benefits and popularity, completion levels are low, which can be attributed to the openness of the environment. In one sense, the far-reaching nature of such courses is an advantage; however, it is also a hindrance as almost anyone can enrol and the consequences for failing are minimal (Balakrishnan and Coetzee 2013).

In order to increase the completion rates of such courses requires insight into potential issues that could hinder a student’s success of finishing their course. However, pinpointing concerns, in a timely manner, becomes harder in an online environment, where the student could potentially be on another continent. In this instance, advanced techniques are required that are able to analyze a student’s online presence and engagement with their course in order to predict their performance so that issues can be flagged up in a timely manner.

### Student engagement and performance in MOOCs

MOOCs attract a wide variety of students, from all over the world and who all have different learning styles. As such engagement, maintaining a level of interest and tailoring the learning environment are more difficult (Chen et al. 2013). As such, in this type of online learning environment, engagement cannot be observed in person and thus becomes more challenging to recognize and measure (Ramesh et al. 2013). For instance, in a classroom setting, if a student is struggling, they have the benefit of building up relationships with their lecturers, who can encourage and talk to them personally about their issues. Furthermore, traditional monitoring mechanisms, such as registers, can be used to pinpoint low attendance, which is linked to poor motivation and performance retention (Field 2012; Muir 2009). As such, issues that could contribute to weak performance and that could be monitored and dealt with in an institution cannot be employed in a distance learning environment. However, by monitoring their online presence, engagement with course materials and online communities could offer an insight into a student’s behavior, which could be used to predict their performance and probability of completion.

Due to the large numbers of participants and complex nature of such courses, the definition of participation and engagement has led to a number of frameworks (Bayne and Ross 2014). For instance, the “funnel of participation,” as described by Clow (2013) attempts to conceptualize the idea of participation into four steps of awareness, registration, activity, and progress. The greatest concentration of students is at the first stage of awareness; as people move through each stage, participation is reduced until only a small number progress and complete the course. In contrast, Kizilcec et al. (2013) categorize learners into patterns of engagement (completing, auditing, disengaging, and sampling). Completing students mirror traditional classroom-based learners and complete the majority of their assessments; auditing learners prefer watching video lectures and completed their assessments infrequently; disengaged students start off strong at the beginning and then decrease their engagement as the course progresses; whilst sampling learners briefly explore the material and preferred to watch videos at the beginning of the course for only a couple of assessments (Kizilcec et al. 2013). Another approach, posited by Hill (2013), offers a similar method of classifying students into five categories (no-shows, observers, drop-ins, passive participants, and active participants). In this study, no-shows appear to be the largest group, with people registering but never logging back in to take part. A trend that has occurred is that all of the groups witnessed a decline in engagement as the weeks progressed (Hill 2013). Meanwhile, Milligan et al. (2013) use a similar approach of three categories of participation (active, lurking, and passive). In their study, “lurkers” seemed to be the largest category of engagement types of learners did follow the course but did not actively engage with other student’s. They preferred to learn independently without communication with the community, such as with the use of blogs or forums. It can therefore be agreed that in order to profile engagement, interaction with course material is vital in understanding the behavior patterns of students. Even though people might not interact with the community, their use of course material still offers a glimpse into their uptake of the course. Furthermore, other avenues, such as blog posts and social media interaction, also pose another interesting line of enquiry to pursue.

Many studies have been undertaken that have explored the use of such variables to determine engagement and performance. For instance, Balakrishnan and Coetzee (2013) used measures including (1) total time spent watching lecture videos, (2) number of threads viewed on forums, (3) number of posts made on forums, and (4) the number of times the course progress page was checked within Hidden Markov Models (HMMs) to study student behavior and retention in MOOCs. This approach was successful in predicting retention and offered an interesting insight into patterns of behavior. For instance, students who rarely or never check their progress, watch no lectures, and do not post/view forums are more likely to drop out (Balakrishnan and Coetzee 2013). In other works, Anderson et al. (2014) have developed a taxonomy of behavior by investigating the role that forum participation plays to the course and by also examining the behavioral patterns of high- and low-achieving students. This work separated students into different engagement styles (viewers, solvers, all-rounders, collectors, and bystanders) by determining the number of assignment questions they attempted and the lectures that they have watched. Furthermore, their final grade is proportional to their activity, with increased interaction with the course (completed assignments, quizzes, viewed lectures, and forum threads) all contributing to a better overall score (Anderson et al. 2014). This work is also of interest as they have tried to increase participation with the introduction of badges as an incentive to participate, with more interaction earning a student more badges. The results concluded that “*making badges more salient produced increases in forum engagement*” (Anderson et al. 2014).

MOOCs are still in their infancy and as with any growing market, they need to ensure that they employ means to maximize their existence in the long term by understanding their customer base (Nawrot and Doucet 2014). Despite their popularity and extraordinary enrolment rates, their high dropout rate is problematic in ensuring this longevity (Nawrot and Doucet 2014). In order to be a viable method of learning, it is therefore vital to increase this completion rate by understanding student engagement in order to minimize dropout rates (Ramesh et al. 2013). As such, interaction with their course is crucial in understanding student behavior so that measures can be employed to reduce the occurrence of dropping out.

### Data analysis techniques in predicting student performance in MOOCs

**Fig. 1:**
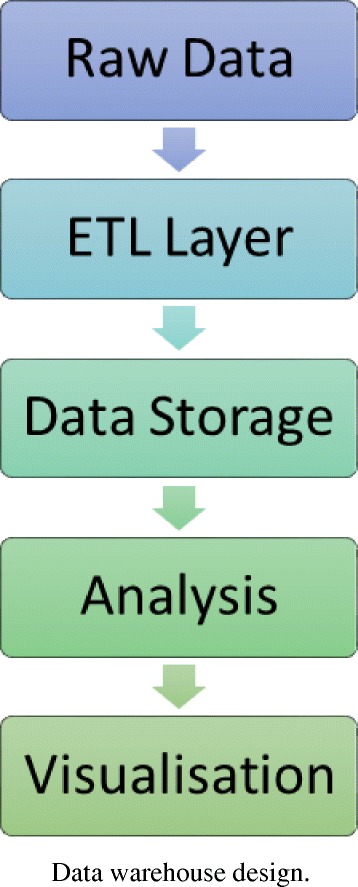
Data warehouse design

Investigating a student’s online behavior and course interaction to predict performance requires sophisticated algorithms and data analysis techniques. One thread of research that is promising in this area is the application of data mining (DM) techniques that are able to turn large datasets into useful information and knowledge (Hanna 2004). Data is being created at a phenomenal rate and can now be stored in many different types of databases, with data warehousing technologies, including data cleansing, integration, and online analytical processing (OLAP), becoming increasingly popular (Hanna 2004). This type of technology is especially useful for mining educational data as it is known for its universality in many applications and for its high performance (Mansmann et al. 2014). Such data warehouses are usually comprised of five layers (see Fig. 1).

**Fig. 2:**
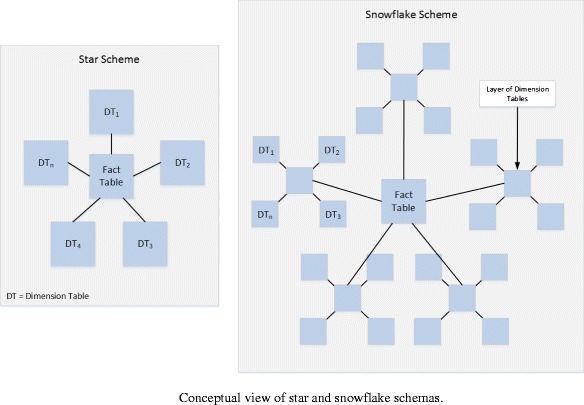
Conceptual view of star and snowflake schemas

In this architecture, raw data is obtained and processed through the ETL (extraction, transform, and load) layer to ensure that its format is compatible before it can be stored in the warehouse. Within this layer, ETL is composed of three stages that are concerned with extracting, transforming, and loading data. The extract stage is concerned with low-level extraction of data from many data sources. These may include databases from numerous commercial vendors (i.e., Microsoft, Oracle, DB2, etc.) or web services, such as RESTful or WSDL based. This data can also be in many formats, including flat file CSV’s (comma-separated file) or semi-structured data such as Extensible Markup Language (XML). Transform refers to a wide ranging set of processes that performs various data operations upon data series such as sorting, grouping, merging, and pivoting data. Typically, the aim of this process is to separate numerical statistics from their textual descriptions. This facilitates the eventual loading of data into structures known as star/snowflake schemas (see Fig. 2).

It is these schemas that form the basis of any Data Warehouse. In Fig. 2, we firstly see the star schema in which a single fact table containing all numerical and summative values resides. Any number of dimensions then describes each row in the fact table. Dimensions typically represent (in the scope of education) dates, courses, modules, topics, etc. The snowflake schema is a logical extension that allows for greater granularity of querying, i.e., instead of just dates, they can be decomposed into years, semesters, weeks, days, etc.

**Fig. 3:**
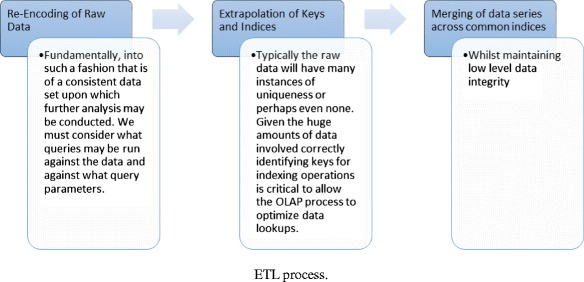
ETL process

In such a system, raw data can be obtained from a range of sources. Interaction with course content, such as lecture videos watched, tests taken, and forum views/posts, can be recorded, as well as personal details (e.g., name, age, gender, and past qualifications) (Hanna 2004; Mostow et al. 2005; Romero et al. 2013). Additionally, activity on blogs, wiki’s, and social media sites are a place where self-directed learners can advance and support their learning and provide a wealth of behavioral data about an individual (Kop and Fournier 2011). In such an environment, analyzing such a heterogeneous set of information requires advanced techniques that can transform this set of raw data into knowledge that can be used to predict performance and to potentially prevent such dramatic dropout figures. In summary, this process requires data to be encoded, extrapolated, and merged into a set of common indices (see Fig. 3).

Once stored, data analysis techniques (OLAP, data mining, etc.) can be employed to obtain knowledge from this information before it is visually communicated to the user (in this case, the student) (Mansmann et al. 2014). Whilst not restricted OLAP, data mining is a common way of categorizing data by identifying patterns that a data series exhibits. The actual methods employed stem from a multidisciplinary arc of computer science and mathematical algorithms. There are several areas that data mining can be employed:

- Anomaly detection: concerned with isolating seemingly erroneous records either for the purpose of anomaly research or correction of errors in the original data series. For example, there are students who defy all preconceptions about the methods of learning yet still succeed or vice versa (Chandola et al. 2009).
- Dependency modeling: concerned with linking knowledge about one data series with knowledge of another. For example, do students spend the same amount of time in study regardless if it be private study or direct contact? (Giraud-Carrier and Povel 2003)
- Clustering: perhaps the most significant, is concerned with normalizing a wide data series into groupings that typically have some association with the mean metric of the group to which they belong (Jain 2010).
- Summarization: refers to the process of summarizing the incoming stream of data or further analysis by transforming raw data into information. For example, we are typically more concerned with the mean and standard deviation, the minimum and maximum, etc. of a set of student’s metrics rather than the raw data itself, though anomalies do need to be examined, as per anomaly detection above (Maimon and Rokach 2010).

As it can be seen, as number of techniques can be employed to predict student performance in an online community. The following sections present an overview of two interesting lines of enquiry, namely machine learning and social media analytics, which utilize varies data mining approaches.

#### Machine learning

As previously discussed, the prevalence of data generation is phenomenal and can be collected from a range of sources, thus producing exabytes of information regularly. However, such streams of information are often unstructured or semi-structured and come from a variety of sources, which makes them more difficult to analyze (Jain 2010). As such, “the increase in both the volume and the variety of data requires advances in methodology to automatically understand, process, and summarize the data” (Jain 2010). This type of data is a fairly recent development in the world of data storage, with the notion of the NoSQL database (Not Only Structured Query Language). These databases do not exclusively rely on the tried and tested models of database design, dating from the 1970s. Instead, they utilize the massive increase in hardware performance to run rapid search/sort and filter algorithms on linear streams of data known as name-value-collections. Examples of such systems are frequently associated with big data analysis and serve to complement rather than replace typical SQL database systems. As such, the area of machine learning is a popular area of research that can be applied to such heterogeneous sets of data to find patterns for predictive modeling, i.e., training data is used to predict the behavior of the previously unseen test data (Jain 2010). This type of learning can either be supervised (classification), where the data is labeled to determine how powerful the algorithm is at learning the solution to the problem, or unsupervised (clustering), where the data is unlabeled and the system forms natural groupings (clusters) of patterns automatically (Duda et al. 2000).

Kloft et al.’s (2014) work uses support vector machines (SVMs) in order to predict when during the course a student will leave. Their work used clickstream data from 3,475,485 web logs from page and lecture video views to train the classifier. The work achieved a moderately good accuracy rate of approximately 72 % at the beginning of the course and this steadily improved over the duration. In other works, Ramesh et al. (2014) use probabilistic soft logic (PSL) to predict whether a learner will complete assignments and quizzes, scoring more than zero, and whether the learner will finish the course. This approach also produced moderately good accuracy rates of 72 % and greater and illustrated that people who were engaged at the start and middle exhibited passive behavior, whilst at the end they become more active (Ramesh et al. 2014).

Jiang et al. (2014) use logistic regression to predict performance using a mixture of a student’s achievement in the first assignment and social interaction within the MOOC community. This work achieved an accuracy of 92 % in predicting whether a student achieved a distinction or normal certificate and achieved 80 % accuracy in predicting whether someone achieved a normal certificate or did not complete (Jiang et al. 2014). In other works, Romero et al. (2013) have developed a data mining tool for Moodle that compares the performance of data mining techniques, including statistical methods, decision trees, rule and fuzzy rule induction methods, and neural networks, to predict a student’s final mark. This work used data from quizzes, assignments, and forums and achieved a very moderate accuracy of 65 %.

In contrast, Ezen-Can et al. (2015) have used an unsupervised clustering approach to gain an insight into the structure of forum posts in MOOCs. The k-medoids algorithm has been used to gain an insight into conversations that learners have on discussion forums. This is an important step in building systems that can automatically understand the topic of the discussion in order to provide adaptive support to individual students and to collaborative groups (Ezen-Can et al. 2015). The literature demonstrates that whilst it is possible to predict student performance from their interaction with course content, further work is required that uses more and different students’ attributes as inputs (Romero et al. 2013).

#### Social media analytics

Social media sites offer a plethora of information about a user, their behaviors and their preferences that can be collected and analyzed. Such outlets are now so pervasive that 91 % of adults use social media and spend more than 20 % of their time on these sites (Fan and Gordon 2014). Additionally, Twitter has 255 million active users who collectively send 500 million tweets per day, whilst Facebook has 1.01 billion mobile monthly active users who have created 50 million pages (Bennett 2014). In order to capitalize on this growth, many companies employ social media analytics to extract useful patterns and intelligence from this data (Fan and Gordon 2014). One key technique in this area is sentiment analysis that can uncover and reveal a variety of behaviors and attitudes of a learner by using text analytics, computational linguistics, and natural language processing to extract emotion or opinion on a subject (Fan and Gordon 2014; Wen et al. 2014).

In one such approach, Wen et al. (2014) have used data from Twitter to study dropout behavior across three MOOCs (teaching, fantasy, and Python courses). In order to achieve this, posts about the specific courses, the lecture topic, and assignments were identified and used in the analysis. The results determined that there was a significant correlation between the mood in the posts and the number of students who drop the course (Wen et al. 2014). In other works, Kop and Fournier (2011) have used blog posts, Twitter, and Moodle participation to identify activities and relationships between learners on the Personal Learning Environments, Networks and Knowledge (PLENK) program; a free course that lasted 10 weeks with 1641 registered participants. Using such data, the findings illustrated that over this period, 900 blog posts and 3104 Tweets were generated; however, regular contributions were only made by 3 % of the group (approximately 40–60 people). The largest group of people were silent and did not produce artifacts nor participate extensively in discussions but they did feel engaged with the course (Kop and Fournier 2011). This study is important as it “provided some clarity on the nature of the interactions between course participants, resources and networks,” whilst highlighting how analytics can be used to understand learners in a distributed, open networked environment (Kop and Fournier 2011).

In other works, Koutropoulos et al. (2014) have analyzed the Twitter stream of a 6-week MOOC and have illustrated that positive emotions were displayed throughout the course and that content was mostly produced during the first few weeks. Furthermore, Twitter itself seems to have been used as an outlet to engage in community learning as participants mainly tweeted to (1) share links containing news and resources, (2) comment about participation or to reflect on learning, or to (3) comment on the live sessions of the course. As such, this data source seems to have become a medium for troubleshooting and broadcasting your activities, outside of the course (Koutropoulos et al. 2014). As it can be seen, social media provides an ideal and open platform to analyze the behavior of learners, outside of the course environment, and provides vital information about behavior and sentiment that should be included when predicting performance. This is useful for predictive modeling where disengaged students can be targeted to ensure that dropout rates do not increase.

### Visualization of data

An often overlooked area of data analysis is the conversion of data into readily readable formats. Many learning analytics solutions are “pedagogically neutral” and do not feature or support formative feedback and simply solely address how educators monitor and provide summative feedback to learner (Alabi et al. 2013). Furthermore, many solutions produce raw data in fantastically un-tabulated/ungrouped data series. However, careful analysis of these results needs presentation, which typically involves transforming their raw data into visually appealing graphs and charts. As such, “Business Intelligence Dashboards” have become a key component in performance management and are a tool to visually summarize large amounts of data (Watson and Wixom 2007). Many implementations exist that can either perform the entire ETL > OLAP > Reporting process or provide front ends to connect to existing OLAP data. Furthermore, such interfaces can display the relevant data to students to indicate their key performance indicators (KPIs) (Golfarelli et al. 2004). For instance, Filva et al.’s study (2014) uses Google Analytics to visualize data about student’s behavior in accessing Moodle content. Data was displayed in a series of graphs, within the dashboard, to illustrate their interaction with the course material. Similarly, Alabi et al. (2013) have visualized learner’s trace data as a timeline that is intended to be a tool to provide formative feedback in order to improve educator efficacy and timely feedback.

In order to effectively communicate a learner’s performance, close attention is required in organizing and displaying such information so that it is useful. If data is not organized efficiently, then it risks becoming meaningless and as a tool to improve performance is useless.

## Methods

**Fig. 4:**
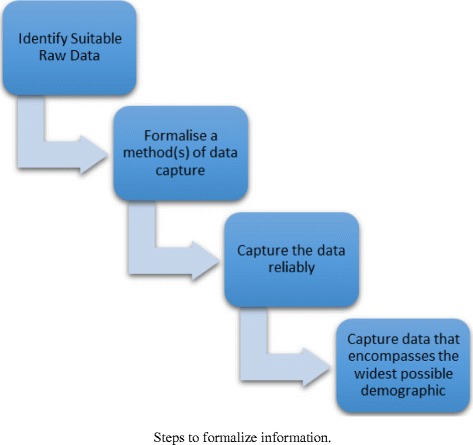
Steps to formalize information

In order to predict a student’s performance to ascertain their probability of completing a MOOC, we first need to address what information is required. To this end, it is necessary to conduct a series of steps to formalize information and the data from which it derives (see Fig. 4).

During this process, identifying suitable data is a human-driven judgement. However, drawing on the literature, it is safe to assume measures such as sex, previous educational qualifications, age, and social media presence, which have been used in previously, are a good starting point (Koutropoulos et al. 2014; Simpson 2006). It is important during the next stage to formalize a method of data capture that is neither controversial ethically or problematic conceptually. In any system that collects and utilizes personal data privacy concerns arise and questions are raised, including “Who keeps and who owns the record of personal preferences? Can individuals view their own records, and what right of response do they have if that information is wrong? What happens if this information is released deliberately or is stolen in a security breach?” (Ashman et al. 2014). These are important points to consider in any system and when addressing such issues, it is important to protect privacy by restricting access to data by adding certification or access control to the data entries and by anonymizing data such that sensitive information cannot be pinpointed to an individual (Wu et al. 2014). A related issue arises in the next stage of data capture as information must be collected reliably to ensure that the mechanisms through which we undertake this collection are secure and deliver unmolested results. Furthermore, in striking a balance between privacy and data, it is also important to capture data that encompasses the widest possible demographic to ensure that we are sampling the breadth of samples as to not distort the results. However, in the experience of the authors, the above is rarely likely to be total in its participation. Indeed, how does one measure a student who does not exist in terms of the metrics defined? Nevertheless, the following approach assumes total engagement with the measured metrics.

### Are we big data?

The authors at this stage avoid the term big data for the purposes of this investigation. Big data is a moniker? When is something broad enough or deep enough to warrant the title “big”? When is data disparate enough to warrant analyses that make it “big”? As such, big data has complicated practical and ethical considerations. For example, if we are to measure every aspect of a student’s engagement in a course, academically and otherwise then the data collected could quite easily be misused for any purposes. Considering the aim is to support student learning by identifying trends, positive or negative, there is only so much that can be done to anonymize the origins of the data. Regardless of what data we capture from what sources, there is typically an issue of the format it is in and whether or not it is fit for purpose in its native condition. More often than not, this will not be the case and it must be pre-processed through refactoring/augmentation either before or during the stages of ETL. Extraction is either a straightforward or a tedious process of accumulating data. One must be careful in assuming that any large dataset is a big data. Big data is an umbrella term, meaningless in itself until it is placed in context. How much data in depth does it take? How much data in breadth does it take? When does one decide this data is big? These are important points to consider when designing any system that requires data to be analyzed to derive meaning.

### Theory-driven vs result-driven analysis

One might wish to pose hypothetical (theory driven) queries to data analysis systems such as “do students who study topics one by one typically perform better than those students who study their topics side-by-side?” In order to answer such a query, there is a predominantly bottom-up process of data analysis, i.e., turning data into information. First, we must isolate the sets that represent polar groupings (clearly being one case or the other) as well as those that lie in groupings somewhere in between. However, on its own this may not be sufficient to produce any firm conclusions. In a system which must analyze many differing metrics, there is a tangible problem of false positives and vice versa. To that end, numerous relatively simple queries should be posed and answered and then their results themselves analyzed in a second round of hypothetical querying.

In contrast, one might wish to identify any commonality between students in a given grouping such as “what characteristics (learning or otherwise) do students who excel at practical topics have?” This type of query can be seen as a more top-down process as we already have the result set but now wish to dig into the metrics that define that set. The issue here is one of metrics explosion as we are attempting to turn information back into data, and there could be a great deal of data to sift through. It is not unreasonable to see if each approach complements the other with one generating information from raw data and the other deriving the raw data that makes up that information.

### Asking correct questions of suitable data

A critical step in the analysis of any data series is to ensure that we firstly know what we are trying to learn or prove/disprove. Secondly, we should be confident that the metrics we are submitting for analysis are actually capable of supporting the derivation of the results we desire. This is not a straightforward requirement as a hypothetical query by its very definition is speculative and the meaning of any results only apparent once they have been generated.

Furthermore, the experimental nature of such data analysis may be prone to the aforementioned false positives and vice versa. When we incorporate a new metric to be measured alongside previously stabled metrics, we need to carefully monitor that new metric’s effect. It will either contradict, reinforce, or have no effect upon the already established patterns.

We must also design in thresholds that cater for anomalies. There will always be a few blocks of raw data that “rock the boat.” When this happens, a choice must be made to exclude them from the overall trend or depending on their frequency of occurrence, perhaps produce additional trends so as to have both conventional data patterns and unusual ones.

### Do we like the results?

Ethical issues perpetually make their presence known, not least in capturing student behavior. In reality, many students may not be overly concerned about monitoring of their learning activity. That said, a spot check by the authors showed that 100 % of students would not like a system to predict their “academic destiny if the outlook was going to be negative.” Rather, do the results of these analysis need to be confined to “need to know” people? Who owns this data; the student or the institution?

## Results and discussion

### eRegister case study

**Fig. 5:**
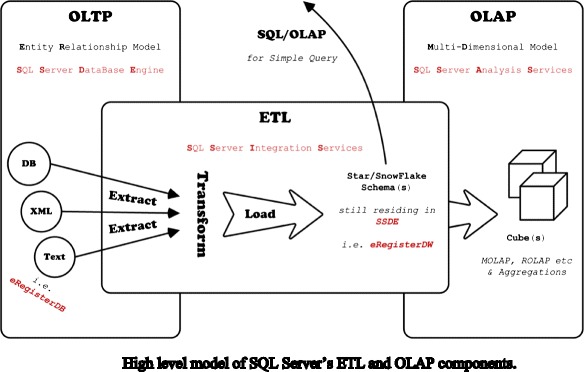
High level model of SQL Server’s ETL and OLAP components

For 7 years, the School of Computing Mathematical Sciences, within the Faculty of Technology and Environment, in Liverpool John Moores University has run an attendance monitoring system, aptly named “eRegister.” The system began life as an exploration into the metrics of students in a controlled group. Over the years, it has grown in depth and breadth. The results it has produced have been interesting, often supporting many well established viewpoints of university learning. Fig. 5 illustrates how eRegister fits into the grander scheme of OLTP, ETL, and OLAP, which all use Microsoft SQL Server as the basis of the data analysis.

Whilst not fitting directly into the MOOC model, the data capture, analysis, and reporting model that eRegister represents is easily extendable into many metrics. As mentioned earlier, the issue of data capture is not as nearly problematic than the analysis of that data. In this scenario, eRegister captures all forms of student attendance (i.e., lecture or lab). Various vectors were employed ranging from direct entry (via eRegister produced printouts), to RFID scanning to post logon Windows NT.x scripts.

As data capture takes place, the database utilizes the process defined in ETL to “fill in the blanks” and normalize the data into a consistent series that is processable together as one result set. The end result is a series of reports that describe course, module, or student attendance.

### Discussion

**Fig. 6:**
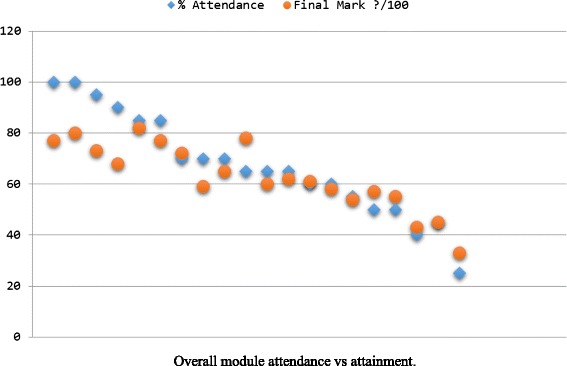
Overall module attendance vs attainment

Recent work has involved placing the reported attendance data, alongside assessment data both in terms of an overall module attendance vs final score as well as trends throughout the year. For example, it was found that students attended more during exam revision periods then they did for coursework revision periods but did not necessarily score better in examinations. As Fig. 6 illustrates, it was however fairly conclusive that attendance does have a real tangible effect on attainment generally. This information could then be discussed with students.

Ideally, students would be able to take a reflective look at their own learning style and make changes should they be needed. By seeing anonymized overall trends, they should be able to identify the simplest areas in which to improve or rather the areas which the trends suggest would allow them to perform to higher standard.

By comparing patterns from year to year, the system would be able to self-evaluate both its effectiveness in highlighting problems and the student’s attempts (or lack of) to rectify those problems through changes in their approach to learning.

This work and the related concepts are easily transferrable to a MOOC environment, where attendance can relate to engagement with lecture videos and assignments, as opposed to physically attending a lecture. As it can be seen, as engagement declines so does the student’ final mark. Using this information, data analysis methods can be employed to predict performance when attendance begins to fall, around 70 %. This would then be visually communicated to the learner that if their current engagement patterns continue, their marks would suffer so that intervention measures can be utilized before attendance drops dramatically.

## Conclusions and future work

The development of the Internet and communication technologies has enabled MOOCs to quickly become a new method for engaging a wider community in open learning. Such developments alter the traditional learning institution paradigm into an open and distance approach, whereby there are no entry qualifications and students study “at their own risk” (Simpson 2006). Nevertheless, in such an environment, it is still important to predict a learner’s chance of success as open institutions have a vested interest in retaining students or risk losing funding (Simpson 2006).

This paper has explored the role that technology can play in open learning to predict a learner’s performance. This is important as identifying “at risk” students before they drop out has the potential to increase MOOC completion rates. As part of the analysis, various areas have been explored, which can be used to predict performance, namely machine learning and social media analytics. The paper has then been concluded with a case study that explores how current techniques, within our institution, can be adapted to such an environment. The eRegister system supports the notion that high/active engagement, interaction, and attendance are reflective of higher marks (Chen et al. 2013; Field 2012; Muir 2009). This work is directly comparable to others in the area. For instance, Balakrishnan and Coetzee’s (2013) approach uses Hidden Markov Models (HMMs) to study student behavior and retention, whilst Anderson et al. (2014) use a taxonomy to define engagement styles. Whilst these have produced good results, they are impractical in the real world. MOOCs contain masses of unstructured data. As this data increases, management is perhaps the biggest problem to address within this paradigm of big data as missing data often occurs and is harder to validate, given the volume of information (Kaisler et al. 2013). Therefore, using HMMs is not applicable as this approach relies on a finite set of data and so cannot be applied if observations are missing (Paroli and Spezia 2002; Van der Heijden et al. 2004). This differs to our work as we have attempted to illustrate how an ETL approach is able to “fill in the blanks” that often occurs within big datasets (Kaisler et al. 2013). Our approach is able to normalize the data into a consistent series so that the end result can be transformed into a dashboard of statistics that can be used by organizers of the MOOC. Although the system has been used within an institution, its relevance within the MOOC community can be seen and as a proof of concept clearly illustrates a need for predictive systems within learning communities.

Future work would consider implementing a version of the eRegister system within a MOOC environment in order to monitor its effect on retention. In this instance, the system could track engagement with course material. A dashboard could also be implemented that would profile an entire course, an individual module, different types of learning activity undertaken as well as individual students so that the lecturer could see how the whole group interacts with the course, as well as the performance/engagement of individuals. For instance, if a student has not been interacting with the course, then the lecturer can be notified so that they can communicate with the student before they disengage completely. Using the system in this way would provide detailed statistics of individuals and would provide an insight into their behaviors.
